# Mild/asymptomatic COVID-19 in unvaccinated pregnant mothers impairs neonatal immune responses

**DOI:** 10.1172/jci.insight.172658

**Published:** 2023-09-12

**Authors:** Brianna M. Doratt, Suhas Sureshchandra, Heather True, Monica Rincon, Nicole E. Marshall, Ilhem Messaoudi

**Affiliations:** 1 Department of Microbiology, Immunology and Molecular Genetics, University of Kentucky, Lexington, Kentucky, USA.; 2 Department of Physiology and Biophysics, School of Medicine, and; 3 Institute for Immunology, University of California, Irvine, California, USA.; 4 Department of Pharmaceutical Sciences, University of Kentucky, Lexington, Kentucky, USA.; 5 Maternal-Fetal Medicine, Oregon Health and Science University, Portland, Oregon, USA.

**Keywords:** COVID-19, Immunology, Cellular immune response, Innate immunity, Monocytes

## Abstract

Maternal SARS-CoV-2 infection triggers placental inflammation and alters cord blood immune cell composition. However, most studies focus on outcomes of severe maternal infection. Therefore, we analyzed cord blood and chorionic villi from newborns of unvaccinated mothers who experienced mild/asymptomatic SARS-CoV-2 infection during pregnancy. We investigated immune cell rewiring using flow cytometry, single-cell RNA sequencing, and functional readouts using ex vivo stimulation with TLR agonists and pathogens. Maternal infection was associated with increased frequency of memory T and B cells and nonclassical monocytes in cord blood. Ex vivo T and B cell responses to stimulation were attenuated, suggesting a tolerogenic state. Maladaptive responses were also observed in cord blood monocytes, where antiviral responses were dampened but responses to bacterial TLRs were increased. Maternal infection was also associated with expansion and activation of placental Hofbauer cells, secreting elevated levels of myeloid cell–recruiting chemokines. Moreover, we reported increased activation of maternally derived monocytes/macrophages in the fetal placenta that were transcriptionally primed for antiviral responses. Our data indicate that even in the absence of vertical transmission or symptoms in the neonate, mild/asymptomatic maternal COVID-19 altered the transcriptional and functional state in fetal immune cells in circulation and in the placenta.

## Graphical abstract

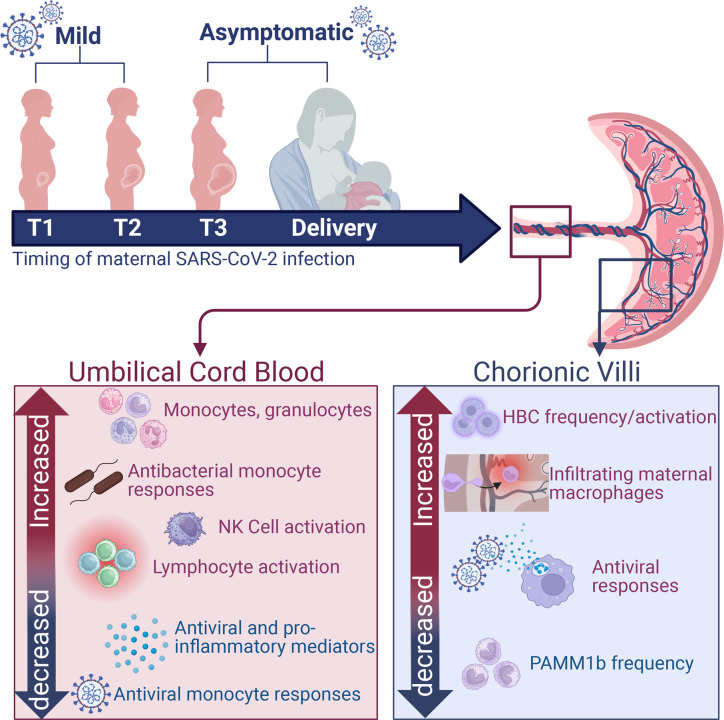

## Introduction

To date, over 250,000 pregnant women in the United States have been infected with SARS coronavirus 2 (SARS-CoV-2) (1). Although most pregnant women experience mild or asymptomatic coronavirus disease 2019 (COVID-19), those who experience severe disease have a higher risk for admission to the intensive care unit, mechanical ventilation, and preterm birth (2, 3). Studies have described the presence of SARS-CoV-2 RNA in placental villi, including within maternal macrophages and Hofbauer cells (HBCs) (4–6). However, vertical transmission is rare (7–10). Nevertheless, SARS-CoV-2 infection during pregnancy alters frequencies of macrophage and effector T cell subsets and induces a pro-inflammatory environment at the materno-fetal interface, specifically within the maternal decidua (11–13). Moreover, pregnant women with severe COVID-19 are more likely to give birth to newborns requiring admission to the neonatal intensive care unit (NICU) with complications associated with preterm birth (14–19). Despite the lack of vertical transmission of SARS-CoV-2, placental inflammation secondary to maternal microbial infection may contribute to fetal immune dysregulation even in full-term, uncomplicated pregnancies, as reported for other maternal infections notably influenza (20, 21), respiratory syncytial virus (RSV) (21), and chorioamnionitis (22, 23).

The mechanistic underpinnings of these adverse outcomes are only beginning to emerge. Recent studies have reported elevated NK cell frequencies in umbilical cord blood (UCB) from neonates born to pregnant women who have recovered from SARS-CoV-2 infection compared with mothers with ongoing infection at delivery (24). Furthermore, UCB NK cells from neonates born to mothers with active SARS-CoV-2 infection or mothers who recovered express higher levels of DNAX accessory molecule 1 (24), an NK cell–activating receptor essential for the recognition and killing of virus-infected cells (25). Mild or asymptomatic maternal SARS-CoV-2 infection detected at delivery also results in an altered inflammatory milieu in fetal circulation, including increased UCB plasma levels of IL-1β, IL-6, IL-8, IL-18, IL-33, IFN-γ, caspase-1, nuclear factor of activated T cells, and CCL21 (13, 26, 27). Additionally, Th2 responses are dampened in infants born to mothers infected during the second and third trimesters (27). Very high anti–SARS-CoV-2 IgG concentrations in UCB were associated with higher frequencies of fetal neutrophils and cytotoxic T cells (26).

Bulk RNA sequencing of UCB cells revealed that mild/asymptomatic maternal SARS-CoV-2 infection in the third trimester is associated with the upregulation of genes responsible for antimicrobial responses and downregulation of genes enriched for phagocytosis, complement activation, and extracellular matrix organization (13). Additionally, UCB monocytes exhibited upregulation of IFN-stimulated genes (ISGs) and MHC class I and II genes (13). Single-cell analysis of UCB from newborns of mothers presenting with mild COVID-19 in the third trimester revealed transcriptional changes indicative of plasmacytoid dendritic cell (pDC) activation, NK cell activation and exhaustion, and clonal expansion of fetal T cells (28). While there are clear disruptions in the UCB immune landscape with maternal SARS-CoV-2 infection, the functional implications of these changes remain largely unknown.

Our previous studies have shown extensive remodeling of decidua (maternal placental compartment) obtained from pregnant women with mild/asymptomatic SARS-CoV-2 infection (11), including altered frequencies of decidual macrophages, regulatory T cells (Tregs), and activated T cells. Furthermore, antigen presentation and type I IFN signaling were attenuated in decidual macrophages, while pathways associated with cytokine signaling and cell killing were upregulated in decidual T cells (11). While abnormal placental pathologies have been reported with maternal SARS-CoV-2 infection, including inflammation and necrosis (29–31), few studies have addressed how maternal SARS-CoV-2 impacts the immune landscape of villous tissues (fetal placental compartment) (13, 32–34). Placental SARS-CoV-2 infection is associated with the recruitment of maternal monocytes and macrophages to villous tissues and increased frequency of fetal HBCs that express programmed cell death ligand 1, a possible mechanism to prevent immune cell–driven placental damage (35). Finally, a recent study reported a downregulation of genes responsible for type I IFN and IL-6/IL-1β cytokine responses in the chorionic villous regardless of maternal COVID-19 severity, the gestational age at infection or delivery, pre-pregnancy body mass index (BMI), or mode of delivery (cesarean versus vaginal delivery) (33).

Despite these observations, our understanding of the impact of mild/asymptomatic maternal SARS-CoV-2 infection on the immune landscape of fetal placental tissues and circulation remains incomplete due to a lack of studies that examined paired samples and where transcriptional analyses were coupled with functional assays. In this study, we used a combination of single-cell RNA sequencing and functional assays to address this gap in knowledge. Our data show that mild/asymptomatic maternal SARS-CoV-2 infection leads to heightened basal activation but dysfunctional responses of both innate and adaptive branches in circulation. This dysregulation extends to the fetal placental compartment (chorionic villi), as shown by the increased infiltration of regulatory maternal monocytes/macrophages into the fetal compartment, HBC activation, and impaired responses of villous myeloid cells to antimicrobial stimulation.

## Results

### Mild/asymptomatic maternal SARS-CoV-2 infection leads to increased systemic fetal inflammation and frequency of myeloid cells.

UCB and placental chorionic villous tissues were collected at delivery from mothers who tested positive for SARS-CoV-2 during pregnancy (mild) or at the time of delivery (asymptomatic) (*n* = 12) and control mothers (*n* = 41) receiving care at Oregon Health and Science University (OHSU). Controls were mostly participants who delivered by scheduled cesarean because of challenges associated with recruitment during the pandemic, hence the higher number of cesarean sections in the control group (67.4%, *P* = 0.0002) (Table 1). Maternal and gestational age at delivery, pre-pregnancy BMI, race, and fetal sex were comparable between both groups (Table 1). Additional cohort characteristics can be found in Table 1.

**Figure 1:**
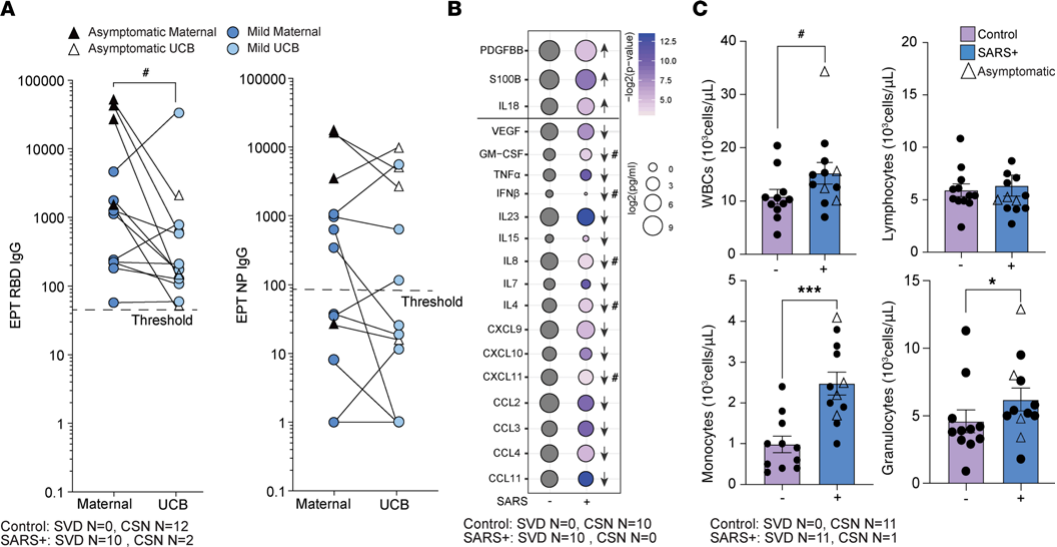
Maternal SARS infection alters frequency of immune cells and immune mediators. (**A**) Maternal and UCB anti-RBD (left) and anti-NP (right) EPTs. *N* = 12/group. (**B**) Bubble plot comparing UCB plasma immune mediators from control and maternal SARS^+^ group. Size represents analyte concentration (pg/mL), whereas color represents statistical significance. *N* = 10/group. (**C**) UCB complete blood cell counts, including white blood cell (top left), lymphocyte (top right), monocyte (bottom left), and granulocyte (bottom right) proportions from control and maternal SARS^+^ groups. *N* = 11 for controls and *N* = 12 for the SARS^+^ group. Group differences between data sets normally distributed were tested using an unpaired *t* test (for data sets with equal variances) or an unpaired *t* test with Welch’s correction (for cases with unequal variances). Data sets not normally distributed were subjected to nonparametric Mann-Whitney test. Error bars represent the data mean ± SEM. (^#^*P* < 0.1, *P < 0.05, ***P < 0.0001.) SVD, standard vaginal delivery; CSN, cesarean section.

All but one of the neonates of participants with SARS-CoV-2 infection had detectable IgG antibodies directed against spike protein RBD at birth, albeit lower than maternal IgG titers (Figure 1A). Additionally, half of the dyads had detectable antibodies against NP, and maternal/neonatal titers were comparable between the 2 groups (Figure 1A). We observed no differences in antibody (IgG) titers against RBD or NP between participants in the mild and asymptomatic groups (Figure 1A and Table 1). Maternal SARS-CoV-2 infection altered immune mediators in UCB relative to control samples, but no differences were observed in UCB immune mediators between the mild and asymptomatic groups (Figure 1B and Supplemental Table 1; supplemental material available online with this article; https://doi.org/10.1172/jci.insight.172658DS1). Specifically, concentrations of several chemokines important for the recruitment of both innate immune cells and lymphocytes (CXCL8, CXCL9, CXCL10, CCL4, CCL3, CXCL11, and CCL11) were lower in the maternal SARS^+^ group (Figure 1B and Supplemental Table 1). Moreover, levels of several antiviral and pro-inflammatory mediators, notably IFN-β, TNF-α, IL-23 (Th17), and IL-15 (NK cell activation), were also lower. Levels of growth factor VEGF, antiinflammatory regulator IL-1RA, and lymphocyte survival factor IL-7 were dampened in the maternal SARS^+^ group. In contrast, levels of S100B, a neurobiochemical marker for CNS injury; PDGF-BB, which regulates cell growth; and IL-18 were increased (Figure 1B and Supplemental Table 1). Moreover, maternal SARS-CoV-2 infection altered the frequencies of circulating white blood cells in UCB, as shown by increased numbers of total white blood cells driven by elevated monocyte and granulocyte numbers (Figure 1C). As described for antibody titers and circulating immune mediators, there was no difference in blood cell counts between the mild and asymptomatic groups.

### Mild/asymptomatic maternal SARS-CoV-2 infection alters the frequency of circulating immune cells, suggestive of a heightened activation state.

**Figure 2:**
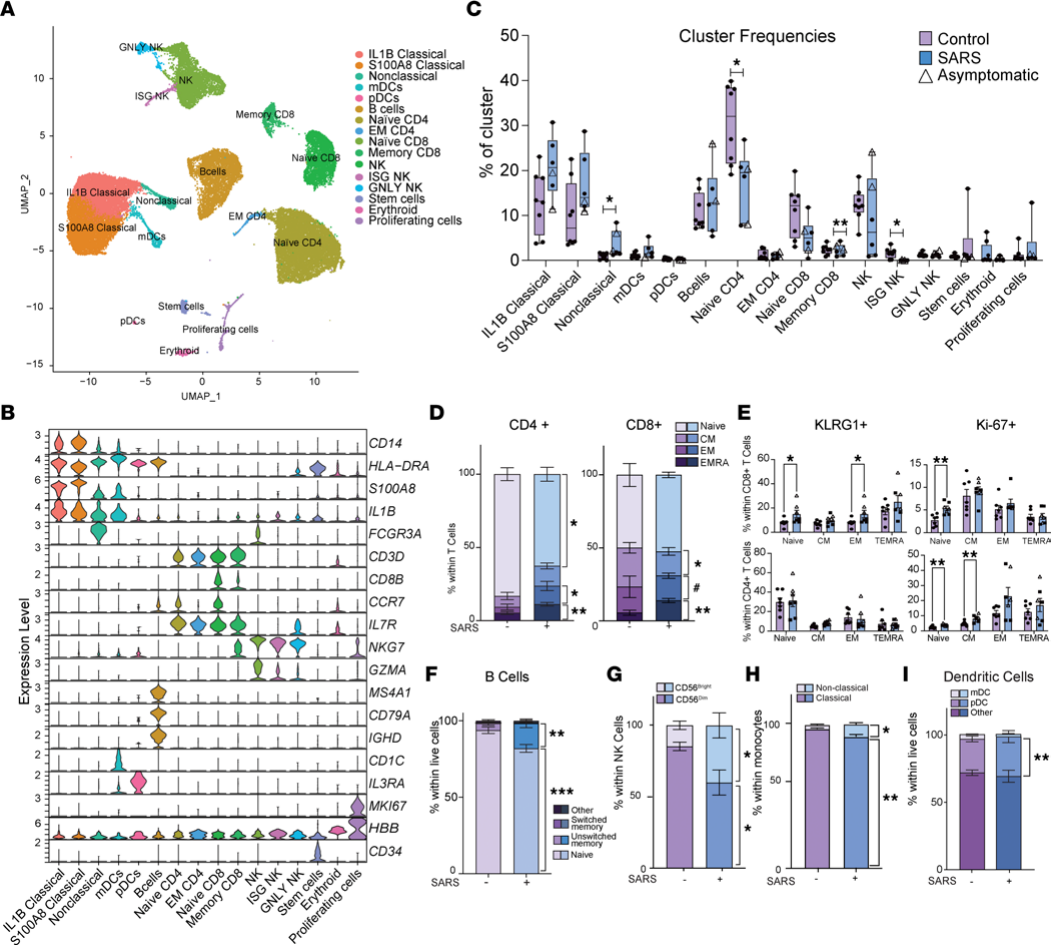
Impact of maternal mild/asymptomatic SARS-CoV-2 infection on phenotype and frequencies of cord blood immune cells. (**A**) Uniform manifold approximation and projection (UMAP) of 42,486 live immune cells from UCBMCs of control and maternal SARS^+^ groups (*N* = 4/group) showing 16 clusters. (**B**) Violin plots of marker genes used for cluster identification. EM, effector memory; GNLY, high expression of *GNLY* gene; mDC, myeloid dendritic cell. (**C**) Box-and-whisker plot comparing cluster frequencies in control and maternal SARS^+^ groups. Triangles indicate asymptomatic maternal infection. Box plots show the interquartile range (box), median (line), and minimum and maximum (whiskers). (**D**) Stacked bar graphs of UCB CD4^+^ and CD8^+^ T cell subset frequencies in control and maternal SARS^+^ groups by flow cytometry. (**E**) Bar graphs comparing KLRG1 and Ki-67 expression within CD4^+^ and CD8^+^ T cells between control and maternal SARS^+^ groups. Triangles indicate asymptomatic maternal infection. (**F**–**I**) Stacked bar graphs comparing (**F**) B cell, (**G**) CD56^bright/dim^ NK cell, (**H**) nonclassical and classical monocyte, and (**I**) dendritic cell subsets between control and maternal SARS^+^ group. For **D**–**I** *N* = 7/group. Group differences between data sets normally distributed were tested using an unpaired *t* test (for data sets with equal variances) or an unpaired *t* test with Welch’s correction (for cases with unequal variances). Data sets not normally distributed were subjected to nonparametric Mann-Whitney test. Error bars represent the data mean ± SEM. (^#^*P* < 0.1, *P < 0.05, **P < 0.01, ***P < 0.001.) CM, central memory.

To uncover the changes within the fetal immune compartment in response to maternal SARS-CoV-2 infection, we performed single-cell RNA sequencing (scRNA-Seq) on UCB mononuclear cells (UCBMCs). We identified 16 unique immune cell clusters (Figure 2A and Supplemental Figure 1A) that were annotated using established gene markers for adult PBMCs (Figure 2B and Supplemental Table 2). Within the lymphoid clusters, B cells were identified based on high expression of *MS4A1*, *CD79A*, and *IGHD*, while T cell subsets were defined based on the expression level of *CD3D*, *CD8B*, *IL7R*, and *CCR7* (Figure 2B). NK cell subsets were identified based on the high expression of *GZMA* and *NKG7* (Figure 2B). Monocyte clusters (classical, intermediate, and nonclassical) were identified based on the expression of *CD14*, *HLA-DRA*, *S100A8*, *IL1B*, and *FCGR3*. Both mDCs (expressing high *CD1C*) and pDCs (expressing high *IL3RA*) were identified (Figure 2B). Additionally, stem cells (expressing *CD34*), proliferating cells (expressing *MKI67*), and a cluster of contaminating erythroid cells (expressing *HBB*) were identified (Figure 2B).

Despite the lack of differences in the total number of circulating lymphocytes (Figure 1C), maternal SARS-CoV-2 infection resulted in decreased frequencies of naive CD4^+^ T cells and NK cells with high ISG signature (Figure 2C). On the other hand, and in line with the increased numbers of circulating total monocytes measured by complete blood cell counts (Figure 1C), the proportion of nonclassical monocytes increased in the maternal SARS^+^ group (Figure 2C). We validated these observations using flow cytometry in a larger number of samples. This analysis verified the reduction of naive CD4^+^ T cells but also revealed a concomitant expansion of both EM and terminally differentiated effector memory (TEMRA) CD4^+^ and CD8^+^ T cells (Figure 2D). Furthermore, expression of the activation marker KLRG1 was elevated in naive and EM CD8^+^ T cells but not CD4^+^ T cells (Figure 2E), whereas expression of the proliferation marker Ki-67 was increased in naive CD4^+^ and CD8^+^ T cells as well as CM CD4^+^ T cells in the maternal SARS^+^ group (Figure 2E). Similarly, a shift from naive to unswitched memory B cell subsets was detected (Figure 2F). Finally, an expansion of immunoregulatory CD56^bright^ NK cells, nonclassical monocytes, and pDCs (Figure 2, G–I) was observed in the maternal SARS^+^ group. No significant differences between mild and asymptomatic SARS^+^ groups were noted (representative example for naive CD4^+^ T cells in Supplemental Figure 1B).

### Mild/asymptomatic maternal SARS-CoV-2 infection results in aberrant activation of fetal lymphocytes.

Given the observed shift toward memory for T and B cells, we used the scRNA-Seq data to interrogate gene expression patterns associated with lymphocyte activation. Within B cells, SARS-CoV-2 infection was associated with increased scores of cytokine signaling and cell migration modules (Supplemental Figure 1C and Supplemental Table 3). Differentially expressed genes (DEGs) with maternal SARS-CoV-2 infection mapped to the regulation of protein kinase activity and immunoglobulin receptor binding Gene Ontology (GO) terms (Supplemental Figure 1D) and included downregulated genes such as *FCRLA*, *MZB1*, *IGLC1/2/3*, *IGKC*, and *CD79B* (Supplemental Figure 1E). Given the downregulation of these key genes, we next tested the impact of maternal SARS-CoV-2 infection on functional B cell responses. Despite the increased frequency of memory subsets, B cells from the SARS^+^ group were less responsive to stimulation with TLR agonist cocktail (36), indicated by lack of induction of CD40 and dampened expression of HLA-DR and costimulatory molecule CD83 (Supplemental Figure 1F).

**Figure 3:**
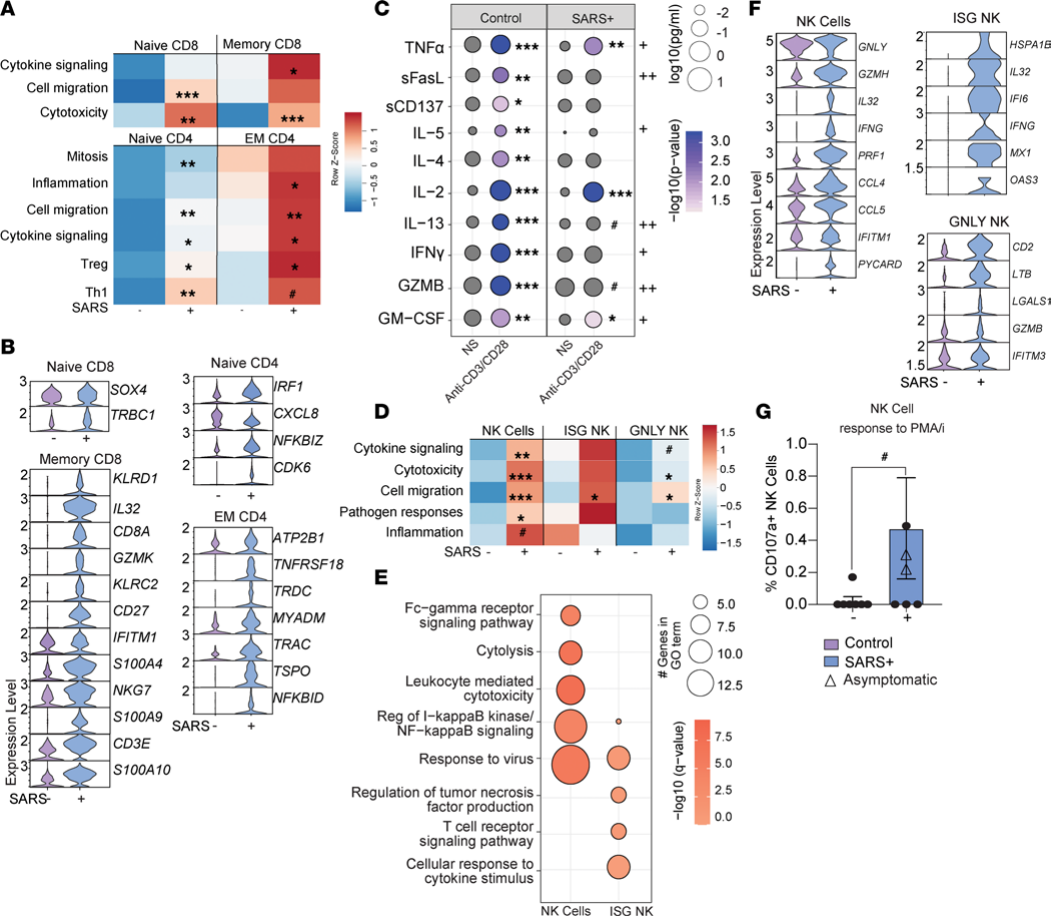
The impact of maternal SARS-CoV-2 infection on fetal lymphocytes and NK cells. (**A**) Heatmap of module scores within T cell clusters for the terms indicated. (**B**) Violin plot comparing normalized transcript counts of select DEGs within the indicated T cell cluster. (**C**) Bubble plot comparing secreted levels of immune mediators in cell culture supernatants following stimulation of UCBMCs from control and maternal SARS^+^ groups with anti-CD3/CD28. Size represents the analyte concentration (pg/mL), and color represents the level of *P* value compared with nonstimulated cells. Plus signs indicate *P* value between stimulated control and maternal SARS^+^ groups (^+^*P* < 0.05, ^++^*P* < 0.01). *N* = 10/group. NS, no stimulation. (**D**) Heatmap of module scores within NK cell clusters for indicated terms. (**E**) Bubble plot comparing functional enrichment of DEGs relative to controls within ISG NK cell and NK cell clusters. Size indicates number of genes and color represents *P* value. (**F**) Violin plot of select DEGs within the shown NK cell clusters. (**G**) Bar graph of total NK cell responses to PMA/ionomycin stimulation. *N* = 7/controls, *N* = 6/SARS^+^ group. Group differences between data sets normally distributed were tested using an unpaired *t* test (for data sets with equal variances) or an unpaired *t* test with Welch’s correction (for cases with unequal variances). Data sets not normally distributed were subjected to nonparametric Mann-Whitney test. Error bars represent the data mean ± SEM. (^#^*P* < 0.1, *P < 0.05, **P < 0.01, ***P < 0.001.)

Within CD8^+^ T cell clusters, there was an increase in transcriptional signatures of cell migration, cytotoxicity, and cytokine signaling with maternal infection (Figure 3A and Supplemental Table 3). DEGs within the memory CD8^+^ T cell compartment were in line with increased potential for cytotoxicity (*IL32*, *GZMK*, *KLRC2*, *KLRD1*, *NKG7*), inflammation (*S100A4*, *S100A9*, *S100A10*), survival/differentiation of activated lymphocytes (*CD8A*, *CD27*, *CD3E*), and antiviral signaling (*IFITM1*) (Figure 3B). Within CD4^+^ T cell clusters, module scores associated with cell migration, cytokine signaling, and Treg and Th1 phenotypes were increased in both naive and EM subsets (Figure 3A and Supplemental Table 3). DEG analysis within naive CD4^+^ T cells revealed increased transcript levels of genes associated with the cell cycle (*CDK6*), consistent with elevated proliferation of naive CD4^+^ T cells in the maternal SARS^+^ group. On the other hand, EM CD4^+^ T cells had increased expression of genes associated with ATP synthesis and mitochondrial homeostasis (*ATP2B1*, *TSPO*) and T cell activation/signaling (*TNFRSF18*, *TRDC*, *TRAC*, *NFKBID*) (Figure 3B).

To interrogate the biological consequences of the changes in activation and transcriptional landscape, UCBMCs from both groups were stimulated with anti-CD3/CD28 beads for 24 hours. T cells from controls generated a robust response as indicated by increased levels of canonical immune mediators (TNF-α, sFASL, sCD137, IL-4, IL-5, IL-2, IL-13, IFN-γ, GZMB, GM-CSF) (Figure 3C). On the other hand, T cells from the maternal SARS^+^ group responded poorly to polyclonal stimulation, indicated by the dampened secretion of Th1 cytokines (IFN-γ, GM-CSF), Th2 cytokines (IL-5, IL-13), and cytotoxic (GZMB) mediators (Figure 3C). These data suggest that heightened maternal inflammation consequent to SARS-CoV-2 infection reprograms neonatal lymphocytes, leading to increased activation at baseline but reduced inability to respond to ex vivo stimulation.

### Mild/asymptomatic maternal SARS-CoV-2 infection enhances fetal NK cell activation.

As described for T cells, maternal SARS-CoV-2 infection was associated with increased scores of modules associated with cytotoxicity, cytokine signaling, cell migration, antiviral and bacterial pathogen responses, and inflammation in NK cell clusters (Figure 3D and Supplemental Table 3). Moreover, gene expression changes in NK cell clusters with maternal SARS-CoV-2 infection mapped to GO terms associated with Fcγ receptor signaling, cytolysis, leukocyte-mediated cytotoxicity, regulation of NF-κB signaling, and viral responses (Figure 3E). This included increased expression of genes such as *GNLY*, *GZMH*, *IL32*, *IFNG*, *PRF1*, *IFITM1*, *IFI6*, *CCL5*, and *PYCARD* across the multiple NK cell subsets (Figure 3F). In line with these observations, an increase in the expression of degranulation marker CD107a by NK cells in response to PMA-ionomycin stimulation was observed in the maternal SARS^+^ group by flow cytometry (Figure 3G), suggesting increased NK cell cytolytic activity. No significant differences were seen in the expression of MIP-1β, IL-2, TNF-α, or IFN-γ by NK cells in response to stimulation (data not shown).

### Myeloid cells from babies born to mothers with mild/asymptomatic SARS-CoV-2 are hyperresponsive to bacterial TLR ligands.

**Figure 4:**
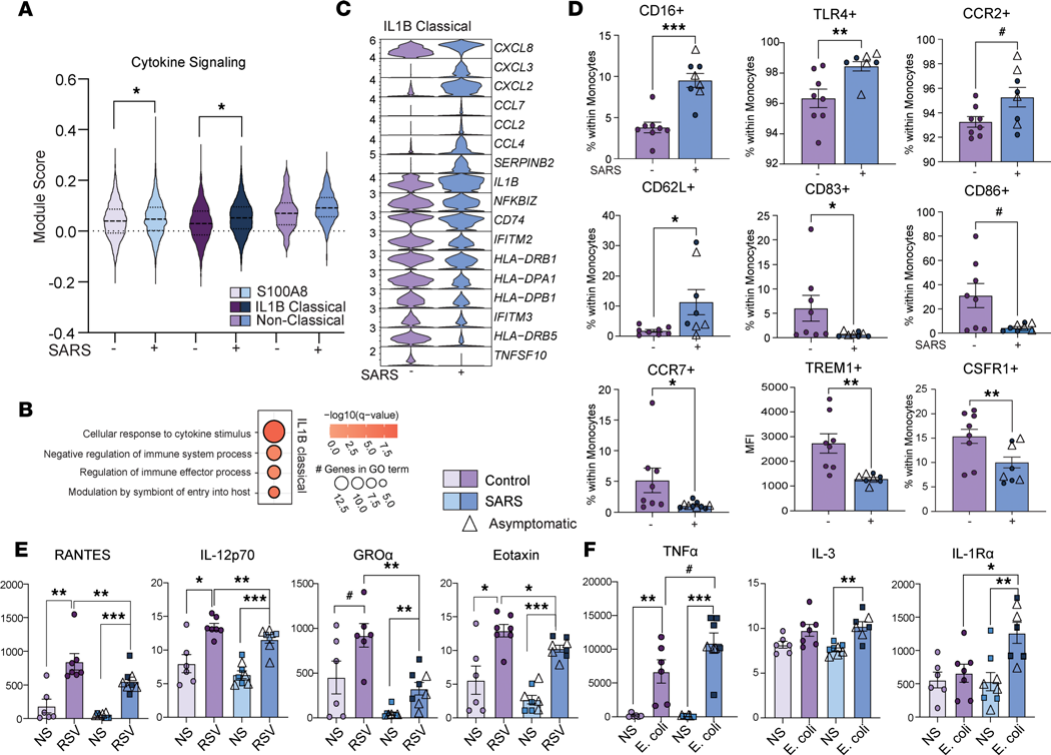
The impact of maternal SARS-CoV-2 infection on fetal myeloid cells. (**A**) Violin plot of module scores within monocyte subsets for cytokine signaling. (**B**) Bubble plot of functional enrichment of DEGs within the IL-1B classical monocyte cluster. Size indicates number of genes and color represents *P* value. (**C**) Violin plots of select DEGs within IL-1B classical monocytes. (**D**) Bar graphs of activation phenotypes by maternal infection status. (**E** and **F**) Scatterplot comparing immune mediators (pg/mL) in culture supernatants of UCBMCs stimulated overnight with (**E**) RSV or (**F**) *E*. *coli* in control and maternal SARS^+^ groups. *N* = 8/group. Group differences between data sets normally distributed were tested using an unpaired *t* test (for data sets with equal variances) or an unpaired *t* test with Welch’s correction (for cases with unequal variances). Data sets not normally distributed were subjected to nonparametric Mann-Whitney test. Error bars represent the data mean ± SEM. (^#^*P* < 0.1, *P < 0.05, **P < 0.01, ***P < 0.001.)

Increased immune activation at baseline was also evident within monocytes as indicated by increased module scores for cytokine signaling in the *IL1B* and *S100A8* classical monocyte clusters (Figure 4A and Supplemental Table 3). Functional enrichment of DEGs revealed an overrepresentation of GO terms associated with responses to cytokines and regulation of immune responses within the *IL1B* cluster (Figure 4B). While chemokine expression was increased in this subset, the expression of MHC class II molecules was reduced in the maternal SARS^+^ group, as was the expression of several ISGs (Figure 4C). These transcriptional patterns suggest a state of immune regulation in monocytes. To test this hypothesis, we assessed markers of monocyte activation using flow cytometry. While expression of CD16, TLR4, and CCR2 was increased in line with immune activation, the frequency of regulatory marker CD62L^+^ increased while that of costimulatory molecules CD83 and CD86, chemokine receptor CCR7, M1-like marker TREM1, and differentiation marker CSF1R decreased on monocytes, indicative of immune regulation (37) (Figure 4D). To test this hypothesis, UCBMCs were stimulated with RSV or *E*. *coli* overnight, and secreted factors were measured using Luminex. While both groups responded to RSV, induction of RANTES, IL-12p70, GROα, and eotaxin was significantly attenuated in the maternal SARS^+^ group (Figure 4E and Supplemental Table 3). In contrast, upon stimulation with *E*. *coli*, secreted levels of TNF-α and IL-1RA were significantly higher in the maternal SARS^+^ group (Figure 4F and Supplemental Table 4). Collectively, these data suggest the rewiring of fetal monocytes toward a state of tolerance to viral antigens but enhanced responses to bacterial ligands.

Finally, within the stem cell cluster, module scores for cytokine signaling, cell migration, and mitosis were increased, suggesting an altered differentiation program (Supplemental Figure 1G and Supplemental Table 3). Interestingly, differential gene expression analysis of the proliferating cell subset showed an overrepresentation of GO terms associated with inflammatory responses, wound healing, and regulation of viral processes (Supplemental Figure 1H), with increased expression of *LYZ*, *CRIP1*, *CD52*, *LGALS1*, and *S100A8*, suggesting that these cells may be myeloid in nature (Supplemental Figure 1I).

### Characterization of the chorionic villous myeloid landscape at term.

**Figure 5:**
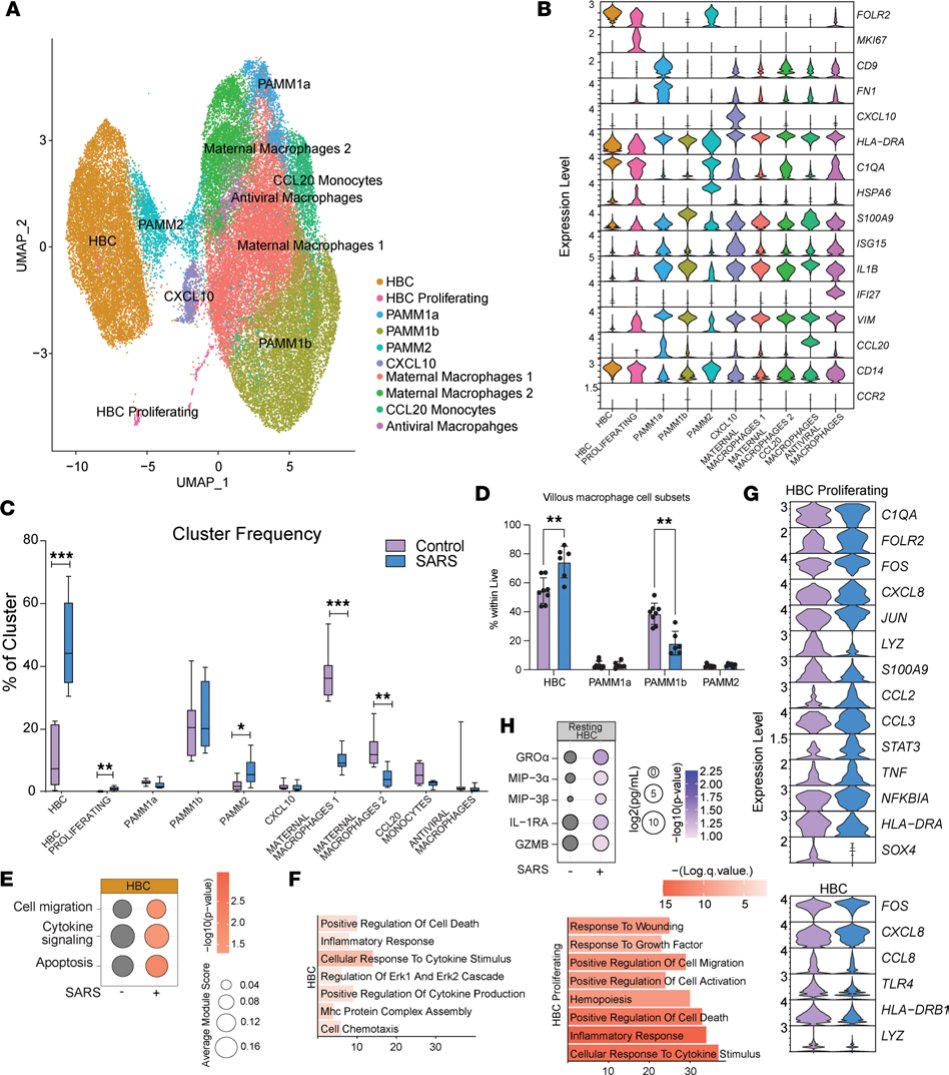
Impact of maternal SARS-CoV-2 infection on immune cells in the villous compartment. (**A**) UMAP of 48,553 immune cells within the villous compartment showing 10 clusters. (**B**) Violin plots of marker genes used for cluster annotation. PAMM, placenta-associated maternal macrophage and monocyte. (**C**) Box-and-whisker plots comparing relative cluster frequencies by infection status. Box plots show the interquartile range (box), median (line), and minimum and maximum (whiskers). (**D**) Bar graph comparing villous monocyte/macrophage subsets identified by flow cytometry. (**E**) Bubble plot comparing module scores within HBC cluster for the terms indicated. Size indicates number of genes and color represents *P* value. (**F**) Bar plot of GO terms for DEGs between controls and maternal SARS^+^ groups from the indicated clusters. Length of the bar indicates the number of genes and color intensity represents *P* value. (**G**) Violin plots of select DEGs within the indicated clusters. (**H**) Bubble plot comparing levels of immune factors secreted by resting HBCs. Size indicates number of genes and color represents *P* value. Group differences between data sets normally distributed were tested using an unpaired *t* test (for data sets with equal variances) or an unpaired *t* test with Welch’s correction (for cases with unequal variances). Data sets not normally distributed were subjected to nonparametric Mann-Whitney test. Error bars represent the data mean ± SEM. (*P < 0.05, **P < 0.01, ***P < 0.001.)

Given the observed changes in circulating fetal immune cells and our recently described changes in decidual leukocytes with maternal SARS-CoV-2 infection (11), we next interrogated the impact of maternal SARS-CoV-2 infection on the immune landscape of chorionic villi (fetal side of the placenta). No viral RNA was detected in any of the villous tissue samples as measured by quantitative PCR (qPCR). Since immune cells in the villi are predominantly myeloid, we sorted CCR2^+^CD14^+^ (monocytes and monocyte-derived macrophages) and CCR2^–^CD14^+^ (tissue-resident macrophages) from villous leukocytes and performed scRNA-Seq on multiplexed controls (*n* = 8) and maternal SARS^+^ samples (*n* = 6). Dimensionality reduction and clustering revealed 10 unique cell clusters that contained cells from both groups (Figure 5A and Supplemental Figure 2A). These clusters were annotated (Figure 5B and Supplemental Figure 2B) based on markers previously described for the first-trimester villous immune landscape (38). HBCs were defined based on high levels of *FOLR2* and low levels of *HLA-DRA*, with a proliferating HBC cluster also expressing high levels of *MKI67*. Placenta-associated maternal macrophage and monocyte (PAMM) clusters were identified based on the relative expression of *CD14*, *CCR2*, *CD9*, *HLA-DRA*, and *FOLR2*. In addition, other maternal infiltrating macrophages were detected and identified based on the relative expression of *HLA-DRA*, *CCL4*, *APOE*, *IL1B*, *CCL20*, *CXCL10*, and ISGs (Figure 5B and Supplemental Figure 2B).

These data provided an opportunity to compare maternal monocytes/macrophages within the decidua (HLA-DR^hi^ and HLA-DR^lo^) (11, 39) and chorionic villous (PAMM1a, PAMM1b, PAMM2, CXCL10^hi^, maternal macrophage 1, maternal macrophage 2, CCL20 monocytes, and antiviral macrophages) at term. To that end, we merged the data obtained in this study with recently described macrophage subsets from matched decidua (11). Dimensional reduction and clustering revealed 5 clusters that contained cells from control and SARS^+^ groups as well as villous and decidua (Supplemental Figure 4, A–C, and Supplemental Table 4). Decidual HLA-DR^lo^ macrophages overlapped predominantly with PAMM1B cells (clusters 0 and 4), while the decidual HLA-DR^hi^ cluster overlapped with the remaining infiltrating villous macrophages (PAMM1a, PAMM2, CXCL10^hi^, maternal macrophage 1, maternal macrophage 2, CCL20 monocytes, and antiviral macrophages) in clusters 1–3 (Supplemental Figure 4, A–C). Decidual macrophages were a major contributor to cluster 1 while the other clusters showed comparable contributions between the 2 compartments (Supplemental Figure 4D). These observations are well aligned with earlier studies that have identified PAMM1b as placental monocytes and PAMM1a as maternal macrophages (38). Collectively, these data support the hypothesis that HLA-DR^lo^ macrophages are recent arrivals into the decidua while HLA-DR^hi^ macrophages are long-term tissue-resident cells, with cluster 3 as the most differentiated. Indeed, expression levels of alarmins (S100A8/A12) and TREM1 were higher in HLA-DR^lo^ and PAMM1b (Supplemental Figure 4E). On the other hand, expression of TREM2 and CD9 was higher in HLA-DR^hi^, PAMM1a, and PAMM2. Additionally, decidual macrophages expressed higher levels of CD68 and VIM but lower levels of LYZ (Supplemental Figure 4E).

We next identified DEGs between decidual and villous cells within each cluster. DEGs upregulated in the villous mapped to oxidative phosphorylation (*COX7C* and *NDUA1*) (Supplemental Figure 4, E and F) and stem cell differentiation while DEGs upregulated in the decidua mapped to hemopoiesis and MAPK signaling (Supplemental Figure 4F). While DEGs from both compartments mapped to terms cellular response to cytokine, regulation of defense response, and other inflammatory processes (Supplemental Figure 4F), the number of DEGs and the significance of the enrichment were higher in the decidua (Supplemental Figure 4F). In line with this observation, expression of several transcriptional factors (*NFKBIA*, *HIF1A*, *FOS*, *JUN*), signaling molecules (*IRAK1*, *PYCARD*), inflammatory mediators (*IL1B*, *TNF*, *C1QA*, *C5AR1*), and genes involved in MHC antigen processing and presentation (*SOCS3*, *CD74*, *HLA-E*, and *HLA-DRA*) was higher in the decidual compartment (Supplemental Figure 4E). These data support the generally held belief that decidual macrophages are the first line of defense against vertical transmission of pathogens.

### Mild/asymptomatic maternal SARS-CoV-2 infection is associated with increased frequency and activation of fetal HBCs.

While maternal SARS-CoV-2 infection was associated with elevated frequencies of resting and proliferating HBCs and PAMM2 cells (infiltrating decidual macrophages), additional subsets of infiltrating maternal macrophages were decreased in the maternal SARS^+^ group (Figure 5C). The increased frequency of HBCs was verified by flow cytometry (Figure 5D and Supplemental Figure 2C). Furthermore, module scores of gene signatures associated with cell migration, cytokine signaling, and apoptosis were elevated in HBCs in the maternal SARS^+^ group (Figure 5E and Supplemental Table 3). Interestingly, DEGs in both HBC subsets in the maternal SARS^+^ group mapped to pathways associated with inflammatory and cytokine responses (Figure 5F). These included both cytokines/chemokines (*CXCL8*, *CCL2*, *TNF*) and canonical transcription factors (*FOS*, *JUN*, *STAT3*, *NFKBIA*) associated with macrophage activation (Figure 5G). To test whether HBCs were activated with maternal SARS-CoV-2 infection, we cultured purified HBCs (CD14^+^FOLR2^+^HLA-DR^–^) for 16 hours and measured secreted levels of cytokines and chemokines at baseline. Indeed, maternal SARS-CoV-2 infection was associated with increased secretion of immune factors associated with myeloid cell recruitment (MIP-3α, MIP-3β) and activation (GROα and IL-1RA) (Figure 5H).

### Single-cell analysis of term chorionic villi reveals adaptations by infiltrating maternal macrophages to mild/asymptomatic maternal SARS-CoV-2 infection.

Flow analyses of macrophage populations within placental villi revealed a decrease in the frequency of PAMM1B cells (maternal monocytes) in the maternal SARS^+^ group, while that of PAMM1a (maternal macrophages) remained unchanged (Figure 5D). However, both populations exhibited altered module scores for antiviral and bacterial defenses, cell and cytokine signaling, and cell migration, apoptosis, and inflammation (Supplemental Figure 2D and Supplemental Table 3). DEGs within the PAMM1a subset mapped to GO terms such as cell activation, cell death/apoptotic signaling, and vessel morphogenesis (Supplemental Figure 2E) and included an increase in the expression of *APOE*, *FN1*, *FCGR2B*, and *JUNB* (Supplemental Figure 2F). On the other hand, DEGs in the PAMM1b subset mapped to GO terms associated with immune activation, cytokine production, and immune effector processes (Supplemental Figure 2E), with upregulation of *ATF4*, *CD55*, *EREG*, *FCN1*, *THBS1*, and MHC class I molecules (*HLA-A*, *HLA-F*) and downregulation of complement transcripts (*C1QA*, and *C1QB*) (Supplemental Figure 2F) in the maternal SARS^+^ group. Finally, while flow analyses revealed no differences in the proportion of PAMM2 cells (Figure 5D), maternal SARS-CoV-2 infection was associated with increased module scores for cell signaling, migration, and inflammation (Supplemental Figure 2D and Supplemental Table 3). Importantly, the maternal SARS^+^ group was linked with downregulation of *IL1B*, *HLA-DRA*, *S100A8/9*, *CXCR4*, *IFI30*, and *TREM1/2* and upregulation of *C1QA*, *CCL2*, and *CSF1R* (Supplemental Figure 2F).

In addition to canonical macrophage populations residing in the placental chorionic villi, we identified additional clusters: a CXCL10^hi^ cluster, 2 maternal macrophage clusters, a CCL10^hi^ monocyte cluster, and an antiviral macrophage cluster (Figure 5B and Supplemental Figure 2B). Maternal SARS-CoV-2 infection was associated with increased cell migration and cytokine signaling module scores in these additional clusters (Supplemental Figure 3A and Supplemental Table 3). A consistent theme across these monocyte/macrophage subsets was the altered expression of genes involved in antimicrobial responses, inflammatory responses, and antigen processing and presentation (Supplemental Figure 3, B and C, and Supplemental Table 3). Gene markers associated with immune activation were elevated in different myeloid subsets — neutrophil chemoattractant *CXCL8* in infiltrating maternal macrophages, ISGs (*IRF1*, *IFI6*) in CCL20 monocytes, and alarmins (*S100A8*, *S100A9*) in antiviral macrophage clusters. We, therefore, posit that an elevated baseline activation state might alter myeloid functional responses to pathogens. We tested this hypothesis by purifying the CD14^+^ cells (all monocytes/macrophages) from chorionic villi and stimulating them with viral and bacterial pathogen-associated molecular patterns (PAMPs). Our analysis of supernatants demonstrated significantly higher levels of pro-inflammatory IL-1α, Flt-3L, and MCP-1 following viral TLR ligand stimulation but no differences in secreted cytokines in response to bacterial PAMPs (Supplemental Figure 3D).

## Discussion

Infectious diseases provoke the maternal immune system (40), which in turn impacts the risk for disease in the offspring (41). Indeed, immune cell ontogeny in early life is particularly vulnerable to maternal infection, as shown by the higher risk for morbidity and mortality from infectious disease in influenza- (42–44), HIV- (45, 46), ZIKV- (47), and malaria-exposed (48) but uninfected infants. Similarly, SARS-CoV-2 provokes maternal immune activation as indicated by increased levels of systemic immune mediators (13, 49). While most studies of COVID-19 in pregnancy have focused on severe cases resulting in fetal distress and preterm birth, there is growing evidence suggesting that mild maternal SARS-CoV-2 alters inflammatory responses at the materno-fetal interface. Therefore, there is a critical need to understand the impact of mild/asymptomatic maternal SARS-CoV-2 infection on the immune landscape of fetal chorionic villous tissues and fetal circulation, specifically in full-term, uncomplicated pregnancies.

Despite the lack of vertical transmission, levels of several chemokines and cytokines necessary for antimicrobial responses were reduced in UCB plasma in the maternal SARS^+^ group. These results indicate that maternal inflammation in the SARS^+^ group may alter immune programming during critical developmental windows for the newborn. Indeed, newborns of mothers with SARS-CoV-2 are more likely to be admitted to the NICU due to complications associated with preterm birth. These observations differ from data reported for nongravid adult SARS-CoV-2 infection, where levels of immune mediators are all elevated (50–52). Our data also differ from those reported in a recent study where a lack of differences, except for a modest increase in IFN-α, in the SARS^+^ group was noted in UCB (53). Interestingly, levels of S100B, IL-18, and PDGFBB, which are linked to neurologic insults in newborns (54–56), were elevated in our study. These data are aligned with increased rates of perinatal brain injury and of neurodevelopmental delays in the first year of life subsequent to maternal SARS-CoV-2 infection (57–59).

As described for adults (60) and infants younger than 1 year of age with mild COVID-19 (61), we report an increased frequency of monocytes and granulocytes in UCB mediated by an expansion of nonclassical monocytes. Although cytokine and chemokine signaling pathways were activated in classical monocyte subsets, expression of HLA-DR and ISGs was reduced in the maternal SARS^+^ group. This is in contrast to earlier studies that reported upregulation of ISGs and MHC genes in UCB monocytes with maternal SARS-CoV-2 infection (28). These discrepancies may be due to the emergence of the more severe Delta SARS-CoV-2 variant during sample collection for this study that was not present when the prior study was completed (28).

Our results show that maternal SARS-CoV-2 infection was associated with the development of “regulatory” monocytes as described for adults with COVID-19 (62–64). Alterations in monocyte activation state may contribute to dysregulated antimicrobial responses. Indeed, UCB monocytes generated an increased response to stimulation with *E*. *coli*, in line with increased expression of the LPS receptor TLR4. However, monocyte responses to RSV were suppressed. We have previously shown the opposite trend with aged adults with COVID-19, where innate immune signaling was preferentially geared toward antiviral responses (64). These observations are aligned with the increased incidence of acute respiratory distress syndrome and pneumonia-like symptoms in newborns of mothers with SARS-CoV-2 during pregnancy (65). This phenomenon is not unique to SARS-CoV-2, since maternal influenza infection during pregnancy results in increased susceptibility of the neonate to viral and bacterial infections (42). Furthermore, maternal inflammation secondary to chorioamnionitis affects neonatal immune programming (66–68), resulting in fetal inflammatory response syndrome (66, 69), an increased risk of chronic inflammatory disorders (68, 70–72), and vulnerability to infection after birth, including early-onset sepsis (73, 74), pneumonia (75), meningitis (76, 77), and necrotizing enterocolitis (78, 79), among others (80). Additional long-term studies will be essential to determine the clinical manifestations of impaired antimicrobial responses in later life with maternal SARS-CoV-2 infection.

Our analysis of UCB revealed accelerated lymphocyte maturation indicated by the increased relative abundance of memory cells and increased expression of the proliferation marker Ki-67 and effector marker KLRG1. Contrary to expectations, stimulation of neonatal T and B cells from the maternal SARS^+^ group resulted in a dampened response to polyclonal stimulation. These observations suggest that these memory lymphocytes may have differentiated in response to maternal inflammation rather than antigen encounter (81). Future studies should address the epigenetic and signaling underpinnings of these expanded yet functionally impaired fetal memory lymphocytes and interrogate their antigen specificities.

We also report a decrease in the frequency of ISG-expressing NK cells but an expansion of cytokine-producing CD56^bright^ NK cells in UCB as well as higher levels of degranulation molecules. These observations align with the increased expression of genes responsible for type II IFN responses and cytolytic functions. Our data are consistent with other studies that reported a decrease in NK cell frequencies but an activated phenotype in adults with SARS-CoV-2 infection as well as in neonates of mothers with SARS-CoV-2 infection during pregnancy (82, 83). Increased activation of fetal NK cells is perhaps a compensatory mechanism for dampened T cell responses.

The human placenta is composed of maternal (decidua) and fetal (chorionic villi) tissues, each with unique immune repertoires (84). The maternal immune cells that populate the decidua include NK cells, macrophages, T cells, and scarce dendritic cells (85), whereas the chorionic villous is composed exclusively of fetally derived macrophages and invading maternal myeloid cells (86, 87). Maternal SARS-CoV-2 infection has been shown to compromise placental function as shown by the increased risk of preeclampsia (88), hypoxia (89), and placental inflammation (90–92). Previous studies, including our own (11), indicate disrupted decidual immune function, including altered antimicrobial function and upregulated cytokine/chemokine signaling in decidual macrophages. Perturbations in maternal circulation and decidual tissues likely expand into fetal villous tissues (93, 94). Indeed, our single-cell and flow cytometry data reveal an increase in the frequency of HBCs with maternal SARS-CoV-2 infection, consistent with previous reports of HBC expansion with adverse pregnancy outcomes (34, 95–97). Furthermore, our analysis indicates increased expression of genes associated with migration, cytokine signaling, and apoptosis in HBCs.

Moreover, pathways associated with cell activation, cell death, and vessel morphogenesis were upregulated in both PAMM1a and PAMM1b subsets. In contrast, expression of several genes involved in host defense and antiviral immunity was decreased in PAMM2 cells. This observation is in line with reports showing dampened expression of genes important for antiviral innate immunity (*IFNB*, *IFIT1*, *MXA*) and cytokine responses (*IL6*, *IL1B*) in chorionic villous tissues by qPCR regardless of gestational age during infection (33, 98). Finally, several additional clusters of infiltrating maternal macrophages were detected in the chorionic villous that highly expressed alarmins, ISGs, *NFKB1*, and MHC class I molecules. Taken together, these findings suggest that maternal SARS-CoV-2 infection triggers different responses within maternal myeloid cells residing in the chorionic villous that are delicately balanced to respond to infection while minimizing inflammation-driven damage to the fetal compartment.

In conclusion, our findings suggest that in the absence of direct infection, maternal inflammation subsequent to SARS-CoV-2 is associated with rewiring of fetal immune cells both in circulation and at the materno-fetal interface. Additional changes were also noted in infiltrating maternal macrophages within the chorionic villous. Importantly, this study highlights that immune adaptations within circulating and tissue-resident fetal myeloid and lymphoid cells can be long-lasting. However, the long-term clinical implications of these changes require additional longitudinal studies of children born to mothers with SARS-CoV-2 infection.

## Methods

### Cohort characteristics.

This study was approved by the Institutional Ethics Review Boards of OHSU and the University of Kentucky. Placental chorionic villi and UCB samples from 41 healthy, pregnant participants without SARS-CoV-2 infection or vaccination who had an uncomplicated, singleton pregnancy and 12 pregnant participants with asymptomatic (*n* = 4) or mild (*n* = 8) SARS-CoV-2 infection, but otherwise healthy pregnancies, were collected. Participants were classified as having mild SARS-CoV-2 infection if they experienced mild respiratory symptoms accompanied by a positive COVID-19 test, while participants were classified as experiencing an asymptomatic infection if they tested positive during the mandatory COVID-19 testing upon admission to labor and delivery and reported no symptoms. Importantly, all nasal swabs from newborns of SARS-CoV-2–infected participants as well as placental chorionic villi tissue samples tested negative for SARS-CoV-2 by qPCR. Controls were participants who did not experience COVID-19 symptoms or report a positive COVID-19 test at any time during their pregnancy who were receiving care at the same facility. The characteristics of the cohort are outlined in Table 1.

### Blood processing.

Whole blood samples were collected in EDTA vacutainer tubes (BD). Complete blood counts were obtained by a Cell-Dyn Emerald 22 (Abbott). UCBMCs and plasma were isolated after whole-blood centrifugation over LymphoPrep in SepMate tubes (STEMCELL Technologies) following manufacturer protocols. Plasma was stored at –80°C until analysis. UCBMCs were cryopreserved using 10% DMSO/FBS and Mr. Frosty Nalgene Freezing containers (Thermo Fisher Scientific) at –80°C overnight and then transferred to a cryogenic unit until analysis.

### Placenta processing.

Fetal chorionic villi were separated from maternal decidua and immediately immersed in RPMI 1640 supplemented with 10% FBS, 1% penicillin-streptomycin, and 1% l-glutamine (GeminiBio). Samples were processed within 24 hours of collection. Chorionic villi were first washed thoroughly in HBSS to remove contaminating blood, then minced into approximately 0.2–0.3 mm^3^ cubes, followed by enzymatic digestion at 37°C for 1 hour in R3 media (RPMI 1640 with 3% FBS, 1% penicillin-streptomycin, 1% l-glutamine, and 1 M HEPES) supplemented with 0.5 mg/mL collagenase IV (MilliporeSigma). The disaggregated cell suspension was passed through tissue strainers to eliminate large tissue chunks. Cells were pelleted and passed sequentially through 100, 70, and 40 μm cell sieves (Falcon, Corning). Red blood cells were lysed using RBC lysis buffer (155 mM NH_4_Cl, 12 mM NaHCO_3_, 0.1 mM EDTA in double-distilled water). The cell suspension was then layered on discontinuous 60% and 40% Percoll gradients (MilliporeSigma) and centrifuged for 30 minutes at 930*g* with the brakes off. Immune cells at the interface of 40% and 60% gradients were collected, counted, and cryopreserved as described above for UCBMCs for future analysis. SARS-CoV-2 viral loads were assessed in placental tissues using qPCR as previously described (11).

### ELISA.

EPTs against the SARS-CoV-2 RBD of the spike protein and NP were determined using standard ELISA as recently described (99). Plates were coated with 500 ng/mL RBD or 1 μg/mL NP (GenScript), and heat-inactivated plasma (1:50 in blocking buffer) was added in 3-fold dilutions. Responses were visualized by adding HRP anti-human IgG (BD Pharmingen clone G18-145) followed by *o*-Phenylenediamine dihydrochloride (Thermo Fisher Scientific). Batch differences were minimized by normalizing to a positive control sample run on each plate. EPTs were calculated using log-log transformation of the linear portion of the curve and 0.1 OD units as the cutoff.

### Plasma luminex.

Levels of immune mediators in plasma, cell culture supernatant following RSV or *E*. *coli* stimulation, and resting HBC culture supernatant were measured using a human, premixed 45-plex panel (R&D Systems). Immune mediators in cell culture supernatant following anti-CD3/CD28 bead stimulation were measured using a human, premixed CD8^+^ T cell 17-plex panel (MilliporeSigma). All Luminex assays were analyzed using a MAGPIX Instrument and xPONENT software (Luminex).

### Phenotyping.

A range of 1 × 10^6^ to 2 × 10^6^ UCBMCs were stained using antibodies against CD4 (BV711, BioLegend, catalog 300558, clone RPA-T4), CD8b (ECD, Beckman Coulter, catalog 6607123, clone 2ST8.5H7), CCR7 (PeCy7, BioLegend, catalog 353226, clone G043H7), CD45RA (PerCPCy5.5, Tonbo Biosciences, catalog 65-040458-t100, clone HI100), CD19 (PE, BioLegend, catalog 302208, clone HIB19), CD27 (APC-Cy7, BioLegend, catalog 356424, clone O323), IgD (AF700, BioLegend, catalog 348230, clone IA6-2), and KLRG1 (APC, BioLegend, catalog 367716, clone SA231a2) to delineate naive and memory T and B cell populations (39). Cells were then fixed (fixation buffer; BioLegend), permeabilized (permeabilization wash buffer; BioLegend), and stained intracellularly for the proliferation marker Ki-67 (FITC, BD Biosciences catalog 556026, clone B56). A second set of samples were stained using antibodies against CD3 (FITC, Tonbo Biosciences, catalog 35-0037-t100, clone OKT3), CD20 (FITC, BioLegend, catalog B218411, clone 2H7), HLA-DR (APC-Cy7, BioLegend, catalog 307618, clone L243), CD14 (AF700, BioLegend, catalog 301822, clone M5E2), CD11c (PEeFluor610, Invitrogen, catalog 61-0116-42, clone 3.9), CD123 (PerCpCy5.5, BioLegend, catalog 30616, clone 6H6), CD56 (BV711, BioLegend, catalog 318336, clone Hcd56), and CD16 (PB, BioLegend, catalog 302032, clone 3G8) to delineate monocytes, mDCs, pDCs, and NK cell subsets (100, 101). All flow cytometry samples were acquired with the Attune NxT instrument (Thermo Fisher Scientific) and analyzed using FlowJo 10.5 (TreeStar).

Villous leukocytes were stained with CD45 (pan-leukocyte marker, BV0605, BioLegend, catalog 304042, clone HI30), CD14 (AF700, BioLegend, catalog 301822, clone M5E2), HLA-DR (APC-Cy7, BioLegend, catalog 307618, clone L243), FOLR2 (APC, BioLegend, catalog 391706, clone 94b/folr2), CD9 (PerCp-Cy5.5, BioLegend, catalog 312110, clone HI9a), and CCR2 (BV421, BioLegend, catalog 357210, clone K036C2) to delineate HBCs (CD14^+^HLA-DR^–^FOLR2^+^CCR2^–^), PAMMs (PAMM1a: CD14^+^HLA-DR^+^FOLR2^–^CD9^+^CCR2^lo/int^) (PAMM1b: CD14^+^HLA-DR^+^FOLR2^–^CD9^–/int^CCR2^+^) and infiltrating maternal decidual macrophages (PAMM2: CD14^+^HLA-DR^hi^FOLR2^hi^) as previously described (87).

### Ex vivo cell stimulation.

For T cell stimulations, 1 × 10^6^ UCBMCs were cultured for 24 hours at 37°C in RPMI supplemented with 10% FBS in the presence or absence of anti-CD3/CD28 beads (Thermo Fisher Scientific). After 24 hours, the cells were spun down, and the supernatants were collected for analysis by Human T-cell 17-plex panel (MilliporeSigma).

For NK cell stimulation, 1 × 10^6^ UCBMCs were stimulated for 6 hours at 37°C in RPMI supplemented with 10% FBS in the presence or absence of 0.5 μg/mL PMA and 5 μg/mL ionomycin (InvivoGen). CD107a antibodies (PE-Cy5, Thermo Fisher Scientific, catalog 15-1079-42, clone eBioH4A3) were added at the beginning of stimulation; Brefeldin A (BioLegend) was added after a 1-hour incubation. Cells were stained for CD3 (BV605, BioLegend, catalog 317322, clone OKT3), CD20 (BV510, BioLegend, catalog 302340, clone 2H7), CD16 (PB, BioLegend, catalog 302032, clone 3G8), CD56 (BV711, BioLegend, catalog 318336, clone Hcd56), and HLA-DR (APC-Cy7, BioLegend, catalog 307618, clone L243); fixed; permeabilized; and stained intracellularly for IL-2 (FITC, BioLegend, catalog 500304, clone MQ1-17H12), TNF-α (APC, BioLegend, catalog 502912, clone Mab11), MIP-1β (PE, BD Biosciences, catalog 550078, clone D21-1351), and IFN-γ (Pe-Cy7, BioLegend, catalog 502527, clone B352664).

For monocytes/macrophages responses, CD14^+^ cells were FACS-sorted from UCBMCs or villous leukocytes and cultured for 16 hours at 37°C in the absence/presence of either RSV (MOI 1) or *E*. *coli* (6 × 10^5^ CFU/well). Production of immune mediators in the supernatants was assessed using a human 45-plex kit (R&D Systems).

### B cell purification and stimulation methods.

B cells were purified from UCBMCs using MACS CD20^+^ microbeads (Miltenyi Biotec). A total of 50,000–100,000 B cells were plated per well and stimulated using a TLR agonist cocktail containing LPS (100 μg/μL), R848 (10 μg/mL), and ODN2216 (5 μg/mL) in RP10 medium (RPMI 1640 with 10% FBS, 1% penicillin-streptomycin, and 1% l-glutamine). Control wells received RP10 + 0.4% DMSO. After stimulation for 24 hours, cells were surface-stained with antibodies against CD3 (BV711, BioLegend, catalog 317327, clone OKT3), CD20 (PB, BioLegend, catalog 302328, clone 2H7), HLA-DR (APC-Cy7, BioLegend, catalog 307618, clone L243), IgD (BV605, BioLegend, catalog 348232, clone IA6-2), CD27 (AF700, BioLegend, catalog 302814, clone O233), CD40 (BV510, BioLegend, catalog 334220, clone 5C3), CD83 (APC, BD Biosciences, catalog 551073, clone HB15e), CD86 (PE, BioLegend, catalog 305406, clone b271481), CD80 (PE-Cy7, BioLegend, catalog 305218, clone 2D10), CD69 (FITC, BioLegend, catalog 310804, clone FN50), and IgG (PECy5, BD Biosciences, catalog 551497, clone G18-145).

### 3′ Multiplexed scRNA-Seq.

Freshly thawed UCBMCs (1 × 10^6^ to 2 × 10^6^ cells) were stained with Ghost Violet 540 (Tonbo Biosciences) for 30 minutes at 4°C in the dark before being incubated with Fc blocker (Human TruStain FcX, BioLegend) in PBS with 1% BSA for 10 minutes at 4°C. Cells were surface-stained with CD45 (FITC, BioLegend, clone HI30) for 30 minutes at 4°C in the dark. Samples were then washed twice in PBS with 0.04% BSA and incubated with individual CellPlex oligos (CMO) (10x Genomics) per manufacturer’s instructions. Pellets were washed 3 times in PBS with 1% BSA, resuspended in 300 μL FACS buffer, and sorted on a BD FACSAria Fusion into RPMI (supplemented with 30% FBS). Sorted live CD45^+^ cells were counted in triplicates on a TC20 Automated Cell Counter (Bio-Rad), washed, and resuspended in PBS with 0.04% BSA in a final concentration of 1,500 cells/μL. Single-cell suspensions were then immediately loaded on the 10x Genomics Chromium Controller with a loading target of 20,000 cells.

Freshly thawed villous leukocytes (1 × 10^6^ to 2 × 10^6^ cells) were stained with Ghost Violet 540 for 30 minutes at 4°C in the dark before being incubated with Fc blocker in PBS with 1% BSA for 10 minutes at 4°C. Finally, cells were surface-stained with HLA-DR (APC-Cy7, BioLegend, catalog 307618, clone L243), CD14 (AF700, BioLegend, catalog 301822, clone M5E2), CCR2 (BV421, BioLegend, catalog 357210, clone K036C2), and FOLR2 (APC, BioLegend, catalog 391706, clone 94b/folr2) for 30 minutes at 4°C in the dark. Samples were then washed twice and incubated with individual TotalSeq B antibodies (HTO) (BioLegend) per the manufacturer’s instructions. Pellets were resuspended in 300 μL FACS buffer and sorted on a BD FACSAria Fusion into RPMI (supplemented with 30% FBS). Sorted live CD14^+^CCR2^+^ and CD14^+^CCR2^–^ cells were counted in triplicates on a TC20 Automated Cell Counter (Bio-Rad), washed, and resuspended in PBS with 0.04% BSA in a final concentration of 1,500 cells/μL. Single-cell suspensions were then immediately loaded on the 10x Genomics Chromium Controller with a loading target of 20,000 cells.

All libraries were generated using the V3.1 chemistry for gene expression and Single Cell 3′ Feature Barcode Library Kit per the manufacturer’s instructions (10x Genomics). Libraries were sequenced on an Illumina NovaSeq 6000 with a sequencing target of 30,000 gene expression reads and 5,000 feature barcoding reads per cell.

### scRNA-Seq data analysis.

Raw reads were aligned and quantified using Cell Ranger (version 6.0.2, 10x Genomics) against the human reference genome (GRCh38) using the *multi-* option. Seurat (version 4.0) was used for downstream analysis. Cell doublets were removed by retaining droplets with a single CMO or HTO signal. Additionally, ambient RNA and dying cells were removed by filtering out droplets with fewer than 200 detected genes and greater than 20% mitochondrial gene expression, respectively. Data objects from controls and SARS^+^ groups were integrated using Seurat. Data normalization and variance stabilization were performed on the integrated object using the *NormalizeData* and *ScaleData* functions in Seurat, where a regularized negative binomial regression was corrected for differential effects of mitochondrial and ribosomal gene expression levels. Dimensionality reduction was performed using *RunPCA* function to obtain the first 30 principal components, and clusters were visualized using Seurat’s *RunUMAP* function. Cell types were assigned to individual clusters using *FindAllMarkers* function with a log_2_ fold-change cutoff of at least 0.4, with FDR < 0.05, and using a known catalog of well-characterized scRNA markers for human PBMCs and villous leukocytes (Supplemental Table 2) (38). Differential gene expression analysis was performed using MAST function in Seurat. Only statistically significant genes maintaining an FDR < 0.05 and a log_2_ fold-change ± 0.25 for UCBMCs or 0.4 for villous leukocytes were included in downstream analyses. Module scores for specific pathways/gene sets were incorporated cluster-wise using the *AddModuleScores* function (Supplemental Table 3). Functional enrichment was performed using Metascape (102).

### Statistics.

Data sets were first assessed for normality using Shapiro-Wilk test and equality of variances using the Levene test. Group differences between data sets normally distributed were tested using an unpaired 2-tailed *t* test (for data sets with equal variances) or an unpaired 2-tailed *t* test with Welch’s correction (for cases with unequal variances). Data sets not normally distributed were subjected to nonparametric Mann-Whitney *U* test. All statistical analyses were conducted in Prism version 9.4.1 (GraphPad). Error bars represent the data mean ± SEM. *P* values less than 0.05 were considered statistically significant.

### Study approval.

Written informed consent from patients for the use of samples for research was obtained at enrollment. This study was approved by the Institutional Ethics Review Boards of OHSU and the University of Kentucky.

### Data availability.

The data sets supporting the conclusions of this article are available on NCBI’s Sequence Read Archive: PRJNA970789, PRJNA970759, and PRJNA847067. Supporting Data Values are available in the supplemental XLS file.

## Author contributions

SS, NEM, and IM conceived the study; SS, NEM, and IM developed methodology; BMD, SS, HT, and NEM investigated; BMD, SS, HT, NEM, and IM wrote the manuscript; NEM and IM acquired funding; and MR and NEM enrolled participants. All authors have read and approved the final draft of the manuscript. The first authorship was determined alphabetically.

## Supplementary Material

Supplemental data

Supplemental table 1

Supplemental table 2

Supplemental table 3

Supplemental table 4

Supporting data values

## Figures and Tables

**Table 1:**
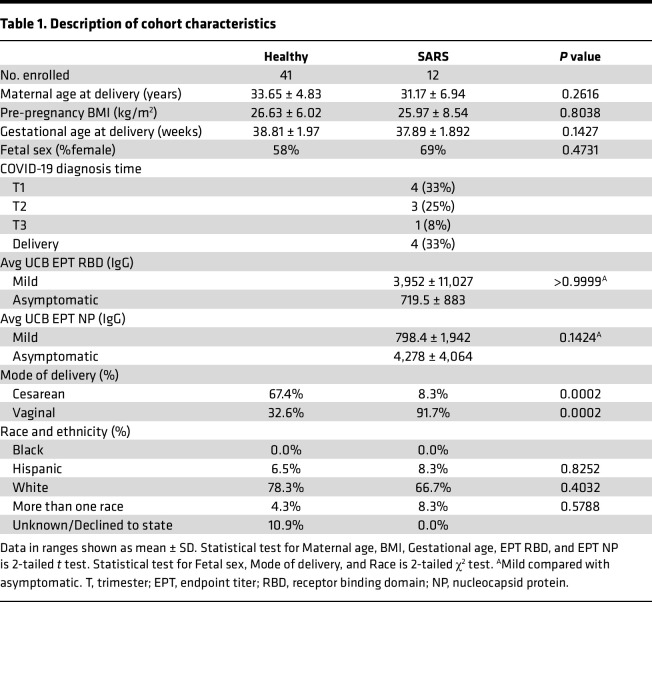
Description of cohort characteristics

## Version 1 09/12/2023

In-Press Preview

## Version 2 10/09/2023

Electronic publication
