# Supplementary material for: Mild/asymptomatic COVID-19 in unvaccinated pregnant mothers impairs neonatal immune responses
Source: JCI Insight. 2023 Sep 12;8(19):e172658. doi: 10.1172/jci.insight.172658 (PMC10629812; doi:10.1172/jci.insight.172658)
Supplement: Supplemental data [file jciinsight-8-172658-s007.pdf]

Supplemental Figure 1

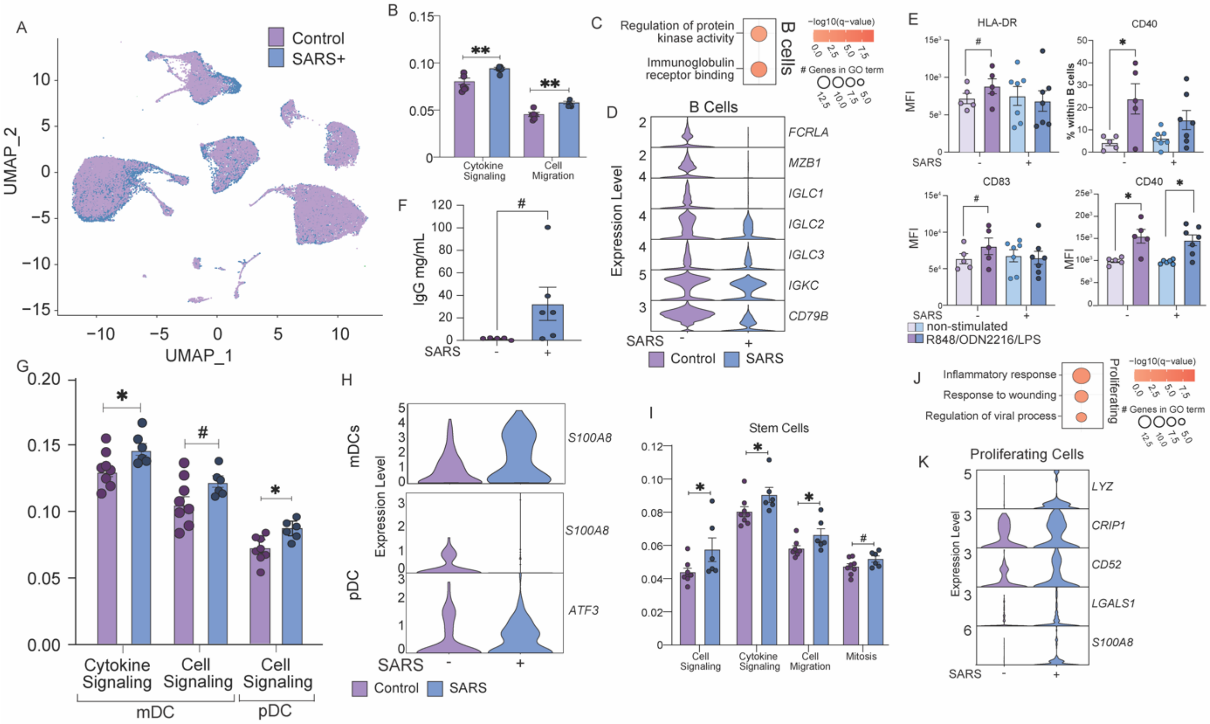

Supplemental Figure 1: UCB Immune cell clusters by infection status and module scores. (A) UMAP of UCB immune cells colored by control (purple) and maternal SARS+ (blue) groups. (B) Bar graph comparing module scores within the B cell cluster for the terms indicated. N=8/group (C) Bubble plot of functional enrichment of top genes within the B cell cluster. The bubble size represents the number of genes mapping to the term, whereas the color represents the level of statistical significance. (D) Violin plot of select statistically significant DEG within the B cell subset. (E) Bar graphs comparing B cell responses to stimulation with R848, ODN2216, and LPS. Triangles indicate UCB samples from mothers with asymptomatic SARS-CoV-2 infection. N=7/group. (F) Bar graph of module scores within the stem cell cluster for the terms indicated. N=8/controls, N=6/SARS+ group. (G) Bubble plot of functional enrichment of top genes within the proliferating cell clusters. The bubble size represents the number of genes mapping to the term, whereas the color represents the level of statistical significance. (H) Violin plot comparing normalized transcript counts of statistically significant DEG within the proliferating cell subset. Group differences between datasets normally distributed were tested using an unpaired t-test (for datasets with equal variances) or an unpaired t-test with Welch's correction (for cases with unequal variances). Datasets not normally distributed were subjected to non-parametric Mann-Whitney test. Error bars represent the data mean ± SEM. (#=p<0.1, *=p<0.05, **=p<0.01, ***=p<0.001).

Supplemental Figure 2:

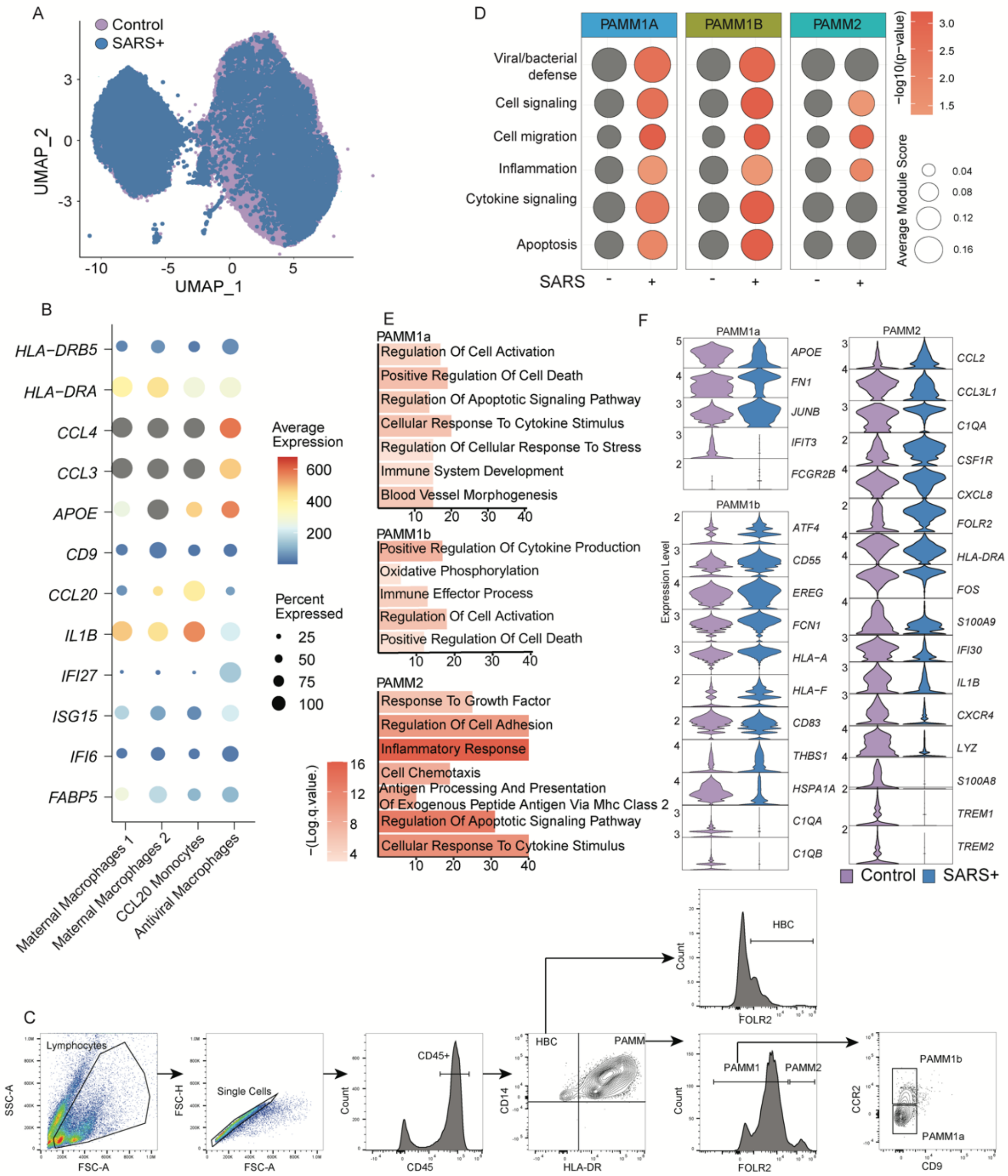

Supplemental Figure 2: Impact of maternal SARS-CoV-2 infection on the immune landscape of the chorionic villous PAMMs. (A) UMAP highlighting villous immune cells from control and maternal SARS+ groups. (B) Bubble plot of additional marker genes used for cluster identification. The bubble size represents the amount of gene expression, whereas color represents the average gene expression. (C) Gating strategy to identify villous immune cell subsets. (D) Bubble plot of module scores within PAMM clusters for the terms indicated. The bubble size represents the average module score, whereas the color represents the level of statistical significance. (E) Barplot of GO terms identified in Metascape for DEG between controls and maternal SARS+ groups from the indicated cluster. Length of the bar indicated the number of genes associated with the term and the color represents the level of statistical significance. (F) Violin plots of select statistically significant DEG within indicated clusters.

Supplemental Figure 3:

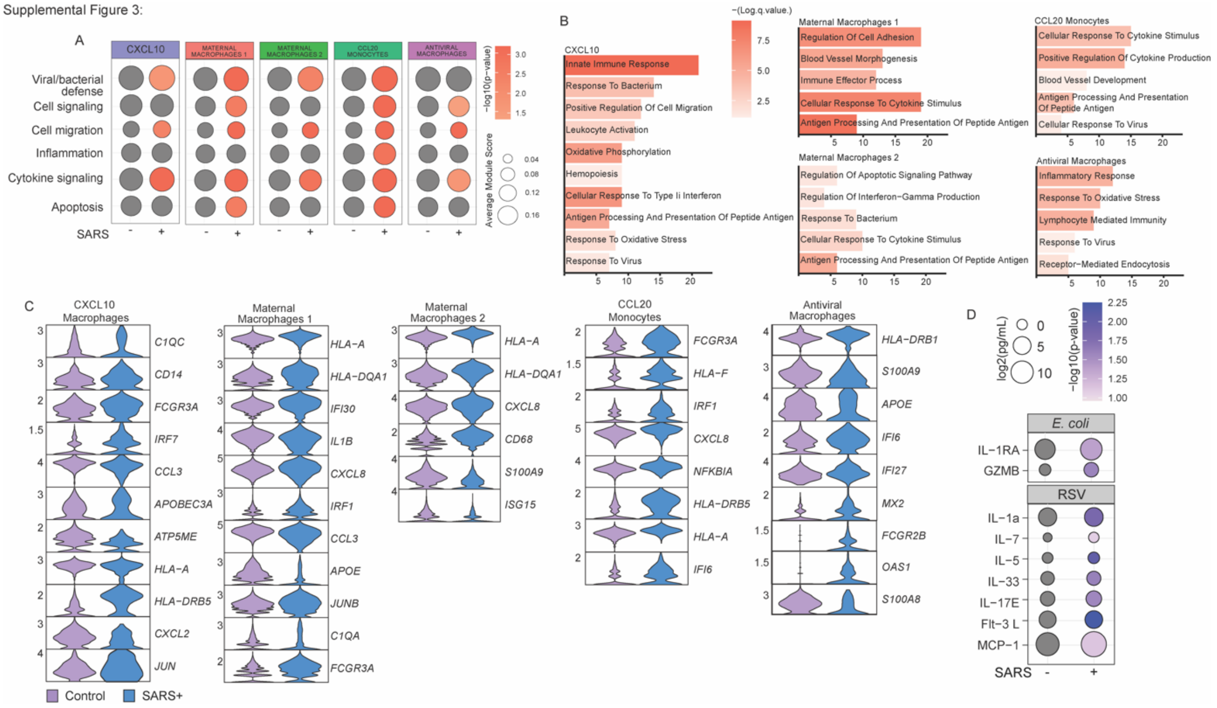

Supplemental Figure 3: Impact of maternal SARS-CoV-2 infection on the immune landscape of the infiltrating myeloid cells in chorionic villi. (A) Bubble plot comparing module scores within infiltrating myeloid cell clusters for the terms indicated. The bubble size represents the average module score, whereas the color represents the level of statistical significance. (B) Bar plot of GO terms identified in Metascape for DEG between controls and maternal SARS+ groups from the indicated cluster. Length of the bar indicated the number of genes associated with the term and the color represents the level of statistical significance. (C) Violin plots of select statistically significant DEG within indicated clusters. (D) Bubble plot comparing immune mediators produced CD45+CD14+ macrophages in response to *E.* coli (top) or RSV stimulation. The bubble size represents analyte concentration, whereas the color represents the level of statistical significance. Group differences between datasets normally distributed were tested using an unpaired t-test (for datasets with equal variances) or an unpaired t-test with Welch's correction (for cases with unequal variances). Datasets not normally distributed were subjected to non-parametric Mann-Whitney test. Error bars represent the data mean ± SEM.

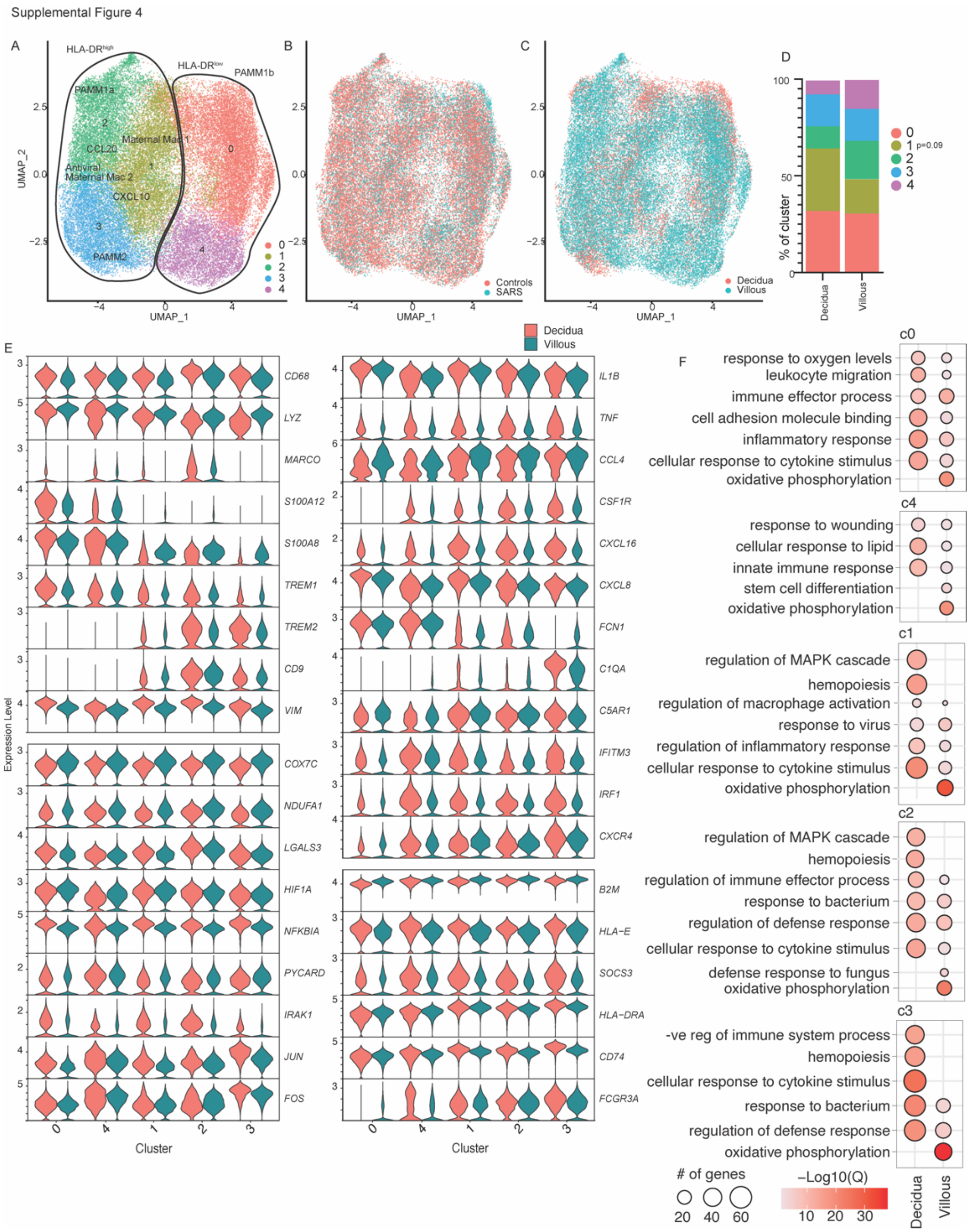

Supplemental Figure 4: Comparison of decidual and infiltrating villi macrophages. (A) UMAP of 41,125 decidual and infiltrating maternal macrophages within the chorionic. The clusters are numbered and colored; the original designation of the infiltrating maternal macrophages/monocyte clusters are indicated in black font; and the two main decidual macrophage clusters are encircled. (B) UMAP from panel A colored by infection condition. (C) UMAP from panel A colored by tissue of origin. (D) Stacked bar graph of cluster frequencies with the decidual and villi compartments. (E) Bubble plots of GO terms to which DEGs between the two compartments within the indicated cluster enriched. The bubble size represents the number of DEGs mapping to the term, whereas color represents the -log10(Q). (F) Violin plots of select statistically significant DEG within indicated clusters in each tissue compartment for all clusters.
